# LC-MS/MS-based metabolomic study provides insights into altitude-dependent variations in flavonoid profiles of strawberries

**DOI:** 10.3389/fpls.2024.1527212

**Published:** 2025-01-07

**Authors:** Muhammad Junaid Rao, Huaizheng Wang, Huaming Lei, Hongcha Zhang, Xiande Duan, Liuyuan Bao, Chengcui Yang, Duo Han, Yongzhi Zhang, Shunqiang Yang, Mingzheng Duan

**Affiliations:** ^1^ College of Agronomy and Life Sciences, Zhaotong University, Zhaotong, China; ^2^ State Key Laboratory of Subtropical Silviculture, College of Forestry and Biotechnology, Zhejiang A & F University, Hangzhou, Zhejiang, China

**Keywords:** strawberry fruit, bioactive flavonoids, fruit quality, antioxidant activities, environment conditions

## Abstract

Environmental conditions significantly influence the metabolic composition and quality attributes of fruits. This study investigated the impact of altitude-associated environmental variation on flavonoid profiles and fruit quality parameters by comparing the “Red Face” strawberry variety grown in two distinct locations: high-altitude-associated environmental conditions in Zhaotong and low-altitude conditions in Dandong. Using LC-MS/MS analysis, we identified 163 bioactive flavonoids, comprising 85 flavonols, 37 flavanones, 33 flavones, and 8 flavanonols. The high-altitude environment of Zhaotong significantly enhanced specific flavonoid compounds, with notable increases in neohesperidin (20.4-fold), tamarixetin-3-O-glucoside-7-O-rhamnoside (17.7-fold), isovitexin (9.1-fold), and hesperidin (8.5-fold) compared to Dandong-grown fruits. Conversely, Dandong-grown fruits showed higher levels of chrysoeriol-7-O-glucoside (53.9-fold), 6-hydroxykaempferol-6,7-O-diglucoside (36.3-fold), and eucalyptin (9.7-fold). The tricetin 3’-glucuronide (24.49% vs 15.31%) and quercetin-4’-O-glucuronide (24.15% vs 15.59%), are the major flavonoids identified in Zhaotong strawberries than Dandong-grown fruits. Furthermore, strawberries cultivated in Zhaotong demonstrated superior antioxidant activities and capacity, increased quality parameters, including higher sugar content (15.30°Brix vs 10.96°Brix), increased ascorbic acid (15.73 mg/g vs 8.53 mg/g), and optimal firmness (20.51 N vs 23.16 N) than Dandong strawberries. These findings suggest that high-altitude cultivation conditions positively influence strawberry fruit characteristics, enhancing both bioactive compound profiles and overall fruit quality. This research provides valuable insights for optimizing strawberry cultivation conditions to maximize nutritional and commercial value.

## 1 Introduction

The strawberries (*Fragaria × ananassa*) are among the most popular and economically significant fruits worldwide, famous for their unique flavor, aroma, and nutritional value. These berries are rich in various bioactive secondary metabolites, which contribute significantly to their health-promoting properties and organoleptic characteristics (Giampieri et al., 2012). Secondary metabolites, particularly flavonoids, have been extensively studied across the plant kingdom, play crucial roles in plant defense mechanisms, fruit quality, and human health benefits (Compean and Ynalvez, 2014; Kabera et al., 2014). Flavonoid-rich fruits demonstrate prolonged beneficial effects on human health (Ghasemzadeh and Ghasemzadeh, 2011). These compounds exhibit significant health-promoting properties, encompassing antioxidants, anti-inflammatory, and anti-tumor activities (Pourcel et al., 2007; Agati and Tattini, 2010; Mathesius, 2018). The flavonoid subclasses, notably flavones and flavonols, demonstrate significant efficacy in mitigating oxidative damage and reducing risks associated with various pathological conditions, including cancer, neurological disorders, and cardiovascular diseases, thereby playing a crucial role in human health maintenance (Cijo et al., 2017; George et al., 2017).

Both flavones and flavonols have been extensively investigated for their robust radical scavenging properties, which not only enable plants to mitigate oxidative stress under adverse environmental conditions but also promote human health by neutralizing reactive oxygen species (Yu et al., 2005; Shen et al., 2022). Research indicates that flavonols effectively inhibit cancer cell proliferation and induce apoptosis, establishing them as promising candidates for cancer prevention and therapeutic interventions. Furthermore, flavonoids enhance cardiovascular function through the improvement of endothelial activity and reduction of blood pressure (Andersen and Markham, 2005). However, the concentration and distribution of these bioactive compounds exhibit substantial variation among plant species, reflecting their distinct genomic profiles (Shen et al., 2022). For example, in strawberries, the biosynthesis of these compounds varies significantly between varieties and cultivars, influenced not only by genetic factors but also by environmental variables (Hanhineva et al., 2008; Giampieri et al., 2012), including growth conditions, abiotic stresses, and agricultural practices.

Altitude-associated ecological conditions, as a key environmental variable, can significantly impact plant metabolism and, consequently, the production of secondary metabolites like flavonoids (Liu et al., 2023). High-altitude environments are characterized by increased UV radiation, lower temperatures, clearer sunshine, and different light/dark durations compared to low-altitude areas. These factors can induce stress responses in plants, potentially altering their metabolic profiles (Ma et al., 2014; Liu et al., 2023). Understanding how high and low altitude-based environmental conditions influence the regulation of flavonol and flavone biosynthesis in strawberries is not only of academic interest but also has practical implications for agriculture, plants, and food science (Mansour et al., 2022). This knowledge can inform breeding programs aimed at developing strawberry cultivars with enhanced flavonoid profiles, improved stress tolerance, and superior nutritional value (Mansour et al., 2022; Liu et al., 2023; Shukla et al., 2024). Moreover, it can guide cultivation practices to optimize bioactive flavonoid compounds in different geographical locations.

Previous studies have demonstrated that environmental factors can significantly affect flavonoid composition in various fruits, including grapes (Mansour et al., 2022), vegetables (Shukla et al., 2024), and potato tubers (Liu et al., 2023). The ‘Red Face’ strawberry variety introduced in 1999 in Dandong city, exhibits superior fruit quality characterized by elevated secondary metabolite concentrations, whereas the ‘Akihime’ variety grown in Zhaotong demonstrates comparatively reduced nutritional composition and metabolite accumulation (Kun et al., 2021). While Zhaotong produces superior quality of apple fruits due to its favorable climatic conditions, particularly significant diurnal temperature variations and high light intensity, strawberry production in this region faces notable challenges (Yuan et al., 2021). The two cultivation sites present substantial geographical contrasts: Zhaotong, situated in southwestern China and Dandong, located in the northeast China. To optimize strawberry production in Zhaotong, this study investigates the feasibility of introducing the Red Face variety. Employing UPLC-MS/MS methodology, we conduct a comprehensive analysis of bioactive flavonoid profiles and assess antioxidant properties under varying environmental conditions. Additionally, we evaluate key quality parameters, including soluble solids content, ascorbic acid concentration, titratable acidity, and fruit firmness. We also examined the altitude-dependent flavonoid accumulation patterns and their correlation with fruit quality metrics. This research will offer valuable insights into our understanding of strawberry adaptation mechanisms to different environmental conditions, particularly focusing on bioactive flavonoid production, and contribute to enhancing strawberry nutritional value through environmental management.

## 2 Materials and methods

### 2.1 Experimental materials and environmental conditions

The “Red Face” strawberry variety was grown in Dandong, northeast China (E 124.23′, N 40.07′, elevation 20 meters above sea level), and was subsequently cultivated in Zhaotong (E 103.72′, N 27.34′, elevation 1900 meters above sea level). For simplicity and clarity, the “Red Face” (*Fragaria × ananassa*) variety Dandong-grown was named as DD99, and cultivated in Zhaotong was named as ZT99, and the indigenous Zhaotong strawberry variety “Akihime” (*Fragaria × ananassa* Duch.) was named as ZT.

Environmental data for both locations was obtained through online link https://globalsolaratlas.info on November 30, 2023. The UV radiation differential between Zhen’an (Dandong) and Zhaoyang (Zhaotong) is attributable to distinct geographical and climatic parameters. Dandong’s higher latitude (40°N) results in reduced UV exposure due to its temperate conditions, while Zhaotong’s lower latitude (28°N) experiences enhanced UV radiation. The substantial altitude differential significantly influences UV intensity, with Zhaotong’s 1900m elevation reducing atmospheric attenuation. Furthermore, Dandong’s humid climate with high precipitation contrasts with Zhaotong’s arid conditions and predominant clear skies, particularly during autumn and winter. Consequently, Zhaoyang experiences elevated UV exposure due to the cumulative effects of elevation, latitude, aridity, and increased solar radiation compared to Zhen’an.

Experimental materials comprised ZT plants obtained from Zhaotong University research plots and DD99 seedlings sourced from Dongji Luyuan Agriculture and Animal Husbandry Co., Ltd. in Fengcheng, Dandong. In August 2023, seedlings approximately 10 cm in height were transplanted under greenhouse conditions. ZT99 and ZT were cultivated at Zhaotong University’s greenhouse facility, while DD99 was grown at Dongji Luyuan’s facility, both under natural environmental conditions following standardized cultivation protocols, optimum fertilization, sandy loam soil, and normal irrigation practices were performed (Reganold et al., 2010; Bottoms et al., 2013). Mature fruit samples were collected simultaneously from all three DD99, ZT99, and ZT samples on December 12, 2023. Each treatment consisted of five biological replicates from individual plants, which were processed via liquid nitrogen grinding, divided into technical triplicates, and preserved in cryotubes. Samples were maintained in liquid nitrogen before transfer to -80°C storage for subsequent UPLC-MS/MS analysis of anthocyanin and proanthocyanidin profiles.

### 2.2 Chemicals

Acetic acid (C_2_H_4_O_2_), acetone (CH_3_)_2_CO, aluminum chloride hexabydrate (AlCl_3_•6H_2_O), 2-(3,4-dihydroxyphenyl)-3,4-dihydro-2H-chromene-3,5,7-triol (catechin C_15_H_14_O_6_), 2,2-diphenyl-1-picrylhydrazyl (DPPH C_18_H_12_N_5_O_6_), ethanol C_2_H_5_OH, methanol (CH_3_OH), hydrochloric acid (HCl), quercetin-3-O-rutinoside (rutin C_27_H_30_O_16_), sodium hydroxide (NaOH), sodium nitrite (NaNO_2_), and 6-Hydroxy-2,5,7,8-tetramethylchroman-2-carboxylic acid (trolox C_14_H_18_O_4_) were purchased from Sigma-Aldrich (Shanghai, China) and quercetin (C_15_H_10_O_7_) was purchased from Xi’an ZB Biotech Co., Ltd., (Shaanxi, China). The metaphosphoric acid (HPO_3_) and 2,6-dichlorophenolindophenol (C_12_H_6_Cl_2_NNaO_2_) were acquired from Suzhou Senfeida Chemical Co., Ltd., (Jiangsu, China) while the L-ascorbic acid (C_6_H_8_O_6_) from Dayang Chem (Hangzhou) Co., Ltd., (Zhejiang, China).

### 2.3 Flavonoid profiling of strawberries

Flavonoid analysis in strawberry fruits was conducted using UPLC-MS/MS methodology. Fruit samples were processed through freeze-drying and pulverization, after which 50 mg of the resultant powder was weighed into tubes for flavonoid analysis. The extraction procedure involved the addition of 500 μL of extraction solution (50% methanol in water containing 0.1% HCl) supplemented with Rutin as an internal standard (4000 nmol/L). The samples underwent ultrasonic extraction for 30 minutes, followed by centrifugation at 12,200×g for 5 minutes at ambient temperature. The supernatant was carefully collected and filtered through a 0.22 μm filter membrane prior to UPLC-MS/MS analysis. Analytical parameters and instrumental configuration were implemented according to established protocol (Chen et al., 2013). Flavonoids identification was achieved through comparative analysis of Q1 precursor ions, Q3 product ions, fragmentation patterns, and peak areas with reference standards under identical analytical conditions, as previously described (Chen et al., 2013). All analyses were performed in triplicate, and mass spectrometric data were processed using Analyst 1.6.3 software. Detailed specifications of the analyzed compounds are presented in **Supplementary Table S1**.

### 2.4 Total flavonoid content

The extraction of total flavonoids was initiated by homogenizing 50 mg of strawberry fruit tissue with 2.5 mL of 80% aqueous methanol solution. Following homogenization, a 0.5 mL aliquot of the supernatant was collected and gently mixed with 2.25 mL of deionized water. The mixture was subsequently treated with 0.15 mL of 5% sodium nitrite solution and incubated at room temperature for 6 minutes. After incubation, 0.3 mL of 10% aluminum chloride hexahydrate solution was added and allowed to react at ambient temperature. The reaction was completed by adding 1 mL of 1M sodium hydroxide, followed by a 2-minute incubation period. Spectrophotometric analysis was performed at 510 nm using a UV-1800 (Shimadzu Corporation, Japan) spectrophotometer (Dewanto et al., 2002). Total flavonoid content was quantified through comparison with a standard rutin calibration curve, and results were expressed as mg/g of rutin.

### 2.5 Sugar and ascorbic acid of strawberry fruits

Total soluble solids (TSS) were measured using a digital refractometer (Magwaza and Opara, 2015). The instrument was calibrated to zero using distilled water prior to analysis. Strawberry juice was obtained through gentle compression of the fruit, and a single drop was placed on the refractometer’s prism. Measurements were performed in triplicate at ambient temperature (21 ± 2°C) and results are presented as Brix.

Ascorbic acid content was determined via titration (Rekha et al., 2012). Sample preparation consisted of homogenizing 10 g of strawberry tissue with 90 mL of 3% metaphosphoric acid solution, followed by filtration. A 10 mL filtrate aliquot was titrated against 2,6-dichlorophenolindophenol until a stable pink coloration persisted for 15 seconds. Vitamin C concentrations were calculated using an L-ascorbic acid standard curve and expressed as mg/g fresh weight (Cordenunsi et al., 2003).

### 2.6 Titratable acidity and firmness of strawberry fruits

Titratable acidity (TA) was assessed by combining 10 g of strawberry puree with 90 mL of distilled water (Cordenunsi et al., 2003). The mixture was titrated with 0.1 N sodium hydroxide to the appropriate pH endpoint. TA percentage was calculated according to the following Equation 1:


(1) TA(%)=(volume of NaOH used × 0.1 N × 0.064 × 100)/sample weight

Where 0.064 represents the citric acid milliequivalent factor.

Tissue mechanical resistance was evaluated using a GY-4 fruit firmness analyzer (GY-4 model, TOP Cloud-agri, Guangzhou, China). Following removal of a small epidermal section, measurements were conducted at two opposing points along the fruit’s equatorial region (Cordenunsi et al., 2003). The maximum force (N) required for a 2 mm compression was recorded using a 4 mm diameter stainless steel cylindrical probe.

### 2.7 DPPH antioxidant activity and capacity

The assessment of antioxidant properties was performed utilizing the DPPH radical scavenging assay (Dudonne et al., 2009). A 0.1 g sample was subjected to extraction using a solvent system comprising ethanol (70%), water (29%), and acetic acid (1%). All reagents required for DPPH analysis were obtained from Sigma-Aldrich. The extraction procedure involved combining 1 mL of the solvent solution with 100 mg of strawberry sample. Following thorough homogenization, the mixture was centrifuged at 12,208×g for 8 minutes. The reaction mixture was prepared by combining 2.97 mL of 0.1 mM DPPH solution with 0.03 mL of the supernatant from the extracted sample. The reaction was allowed to proceed under dark conditions for 30 minutes, after which absorbance measurements were recorded at 517 nm using a UV-1800 spectrophotometer. A control solution was prepared following the same protocol, substituting the sample extract with deionized water (0.03 mL). Results were expressed as millimolar trolox equivalents per 100 mg sample. The radical scavenging activity was calculated according to the following Equation 2:


(2) Antioxidant activity(%)= 100 × [1−(Absorbance sample/Absorbance control)]

### 2.8 Ferric reducing antioxidant power capacity

The antioxidant capacity of strawberry fruits was assessed utilizing the Ferric Reducing Antioxidant Power (FRAP) assay (Benzie and Strain, 1996). The FRAP reagent was prepared by combining three components in a 1:10:1 ratio: ferric chloride hexahydrate (FeCl_3_•6H_2_O, 20 mmol in 40 mmol/L HCl), acetate buffer (300 mmol, pH 3.6), and 2,4,6-tris(2-pyridyl)-s-triazine (TPTZ) solution (100 mmol/L in 40 mmol/L HCl). For the analysis, strawberry fruit tissue samples (100 mg) were homogenized, and an aliquot of 25 μL supernatant was combined with 175 μL FRAP reagent. The mixture was incubated at room temperature in darkness for 30 minutes. The absorbance of the resultant blue Fe^2+^-TPTZ complex was measured spectrophotometrically at 593 nm using a UV-1800 spectrophotometer. A blank measurement was conducted using acetate buffer as a reference. The final FRAP value was calculated by subtracting the blank absorbance from the sample absorbance. This methodology quantified the antioxidant potential through the assessment of ferric ion reduction capacity, with results expressed as mmol Fe^2+^ per gram of strawberry fruits tissue (Benzie and Strain, 1996).

### 2.9 2,2′-azinobis (3-ethylbenzothiazoline-6-sulphonic acid radical cation (ABTS•+) radical scavenging assay

The antioxidant activity was evaluated utilizing an adapted protocol based on the ABTS radical scavenging method (Dudonne et al., 2009). The ABTS•+ stock solution was prepared by combining equimolar volumes of ABTS (7 mmol/L) and potassium persulfate (2.45 mmol/L) solutions. The reaction mixture was incubated in darkness at an ambient temperature for 12 hours to ensure complete radical generation. Subsequently, the stock solution was diluted with methanol to achieve an absorbance of 0.70 ± 0.02 at 734 nm, measured using a UV-1800 spectrophotometer. The experimental protocol consisted of combining 2 mL of ABTS•+ solution with 1 mL of sample extracts at concentrations ranging from 0.5-5.0 mg/mL. The mixture was incubated under dark conditions at room temperature for 10 minutes. A control was prepared by combining 2 mL ABTS•+ solution with 1 mL double-distilled water, while butylated hydroxytoluene (BHT) was used as the reference standard. Absorbance measurements were conducted in triplicate at 734 nm against a blank using the UV-1800 spectrophotometer. The radical scavenging capacity was calculated using the following Equation 3:


(3) Scavenging percentage (%)=[(Control absorbance − Sample absorbance)/Control absorbance] × 100

### 2.10 Statistical analysis

Metabolomic data underwent Z-score normalization procedures prior to hierarchical cluster analysis (HCA). The statistical framework encompassed HCA and principal component analysis (PCA), following standardized protocols outlined in reference (Mu et al., 2024). Graphical visualizations and standard error computations were executed using Microsoft Excel software. The least significant difference test was utilized to assess variations in antioxidant concentrations and total flavonoid levels across different strawberry varieties. All statistical analyses of antioxidant activities and flavonoids content were performed using Statistix 8.1 software, with triplicate biological samples.

## 3 Results

This study utilized LC-MS/MS to elucidate the complex flavonoid profile of strawberry fruits across various varieties grown under different altitude-associated environmental conditions. The widely targeted metabolomics approach identified 163 distinct bioactive flavonoids, categorized into four subclasses: 85 flavonols, 37 flavanones, 33 flavones, and 8 flavanonols (**Supplementary Table S1**). For each identified flavonoid, we documented the molecular nature and chemical properties, including molecular weights, chemical formulas, structural classifications, Q1 and Q3 values related to mass spectrometric analysis, and ionization modes (**Supplementary Table S1**). To facilitate cross-referencing with established chemical databases, Chemical Abstracts Service (CAS) registry numbers and KEGG compound IDs were also recorded for the identified flavonoid (**Supplementary Table S1**).

### 3.1 Flavonols regulation in strawberries cultivated in varying environments

**Figure 1:**
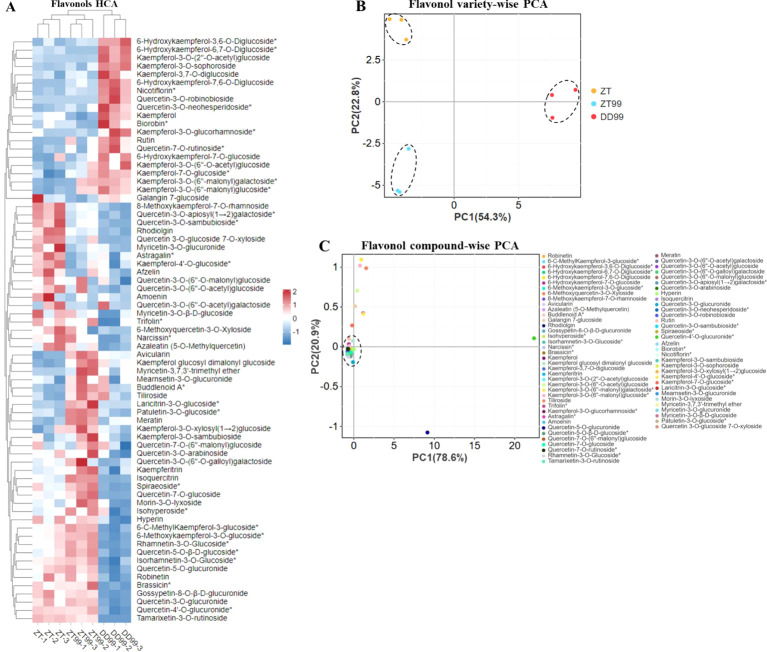
Hierarchical cluster analysis (HCA) and principal component analysis (PCA) of flavonols in strawberry fruits cultivated under varying environmental conditions. **(A)**: Flavonols HCA, the rows denote the flavonols, and the column illustrates the strawberry varieties, rows were normalized, **(B)**: Flavonols strawberry variety-wise PCA, and **(C)**: Flavonols compound-wise PCA.

Hierarchical cluster analysis (HCA) was achieved to assess the variation and abundance of flavonols across different strawberries grown in diverse environments (**Figure 1A**). The analysis revealed that the DD99 variety, grown in a lower-altitude environment, formed a distinct cluster compared to ZT99 and ZT variety grown at higher altitudes (**Figure 1A**). This finding indicates that altitude-ecological conditions significantly influence flavonol biosynthesis in strawberry fruits. Approximately 20 flavonols exhibited higher abundance in the DD99 variety compared to ZT and ZT99 (**Figure 1A**). This observation underscores the significant impact of diverse ecological conditions on the synthesis of beneficial bioactive compounds within the plants. The ZT variety demonstrated a higher abundance of 18 flavonol compounds, while ZT99 showed a higher abundance of 33 flavonols compared to DD99, and 24 flavonols were more abundant in ZT99 fruits than in the ZT variety (**Figure 1A**). Notably, cultivation of the “Red Face” strawberry, introduced from Dandong to Zhaotong resulted in a marked increase in the concentration of several health-promoting flavonols in ZT99 fruits compared to the native Zhaotong ZT variety.

The variety-wise principal component analysis (PCA) revealed distinct separations of ZT, ZT99, and DD99 varieties (**Figure 1B**). The ZT variety clustered in the upper-left quadrant of the PCA plot, while DD99 was positioned on the right side on the x-axis. The ZT99 was located in the lower-left quadrant (**Figure 1B**). This finding highlights the substantial influence of diverse environmental conditions on the production of these beneficial compounds within the strawberry fruits. PC1 accounted for 54.3% of the variation along the x-axis, while PC2 explained 22.8% of the variation. These results indicate significant variations in the flavonol profiles among the three strawberries.

Compound-wise PCA analysis demonstrated that the majority of compounds clustered at the intersection of the x- and y-axis, with the exception of 10 flavonol compounds (**Figure 1C**). These 10 flavonols were distinctly separated from the main cluster and accounted for the maximum variation in both PC1 and PC2. PC1 explained 78.6% of the variation along the x-axis, while PC2 accounted for 20.9% of the variation along the y-axis. These findings suggest that strawberry plants undergo biochemical adaptations in response to varying growth environments, potentially enhancing their nutritional value and pharmaceutical applications.

### 3.2 Clustering and grouping of flavonoids in strawberry fruits

**Figure 2:**
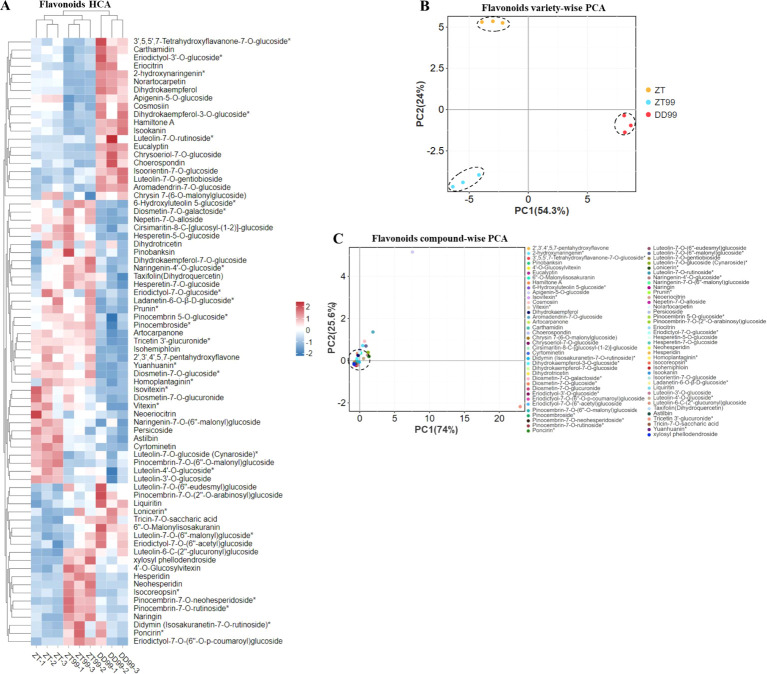
HCA and PCA of flavonoid compounds in strawberry fruits cultivated under varying environmental conditions. **(A)**: Flavonoids (including flavanones 37, flavones 33, and flavanonols 8) HCA, the rows signify the flavonoid compounds, and the column characterizes the strawberry varieties, rows were normalized, **(B)**: Flavonoids variety-wise PCA, and **(C)**: Flavonoids compound-wise PCA.

A comparative analysis of flavonoid profiles (including 37 flavanones, 33 flavones, and 8 flavanonols) in strawberries cultivated under varying environmental conditions was conducted using HCA (**Figure 2A**). The results reveal distinct patterns in flavonoids abundance and distribution across different strawberry varieties. Similar to flavonol HCA analysis, the flavonoids profiles of DD99 exhibited a unique clustering pattern when compared to the ZT99 and ZT varieties (**Figure 2A**). The HCA analysis identified approximately 28 flavonoids that were more prevalent in the DD99 variety compared to ZT and ZT99 varieties (**Figure 2A**). Furthermore, the ZT variety showed elevated levels of 13 flavonoid compounds than DD99 and ZT99, while the ZT99 strawberry demonstrated higher concentrations of 43 flavonoids relative to DD99. Additionally, 22 flavonoids were found to be more abundant in ZT99 fruits compared to the ZT (**Figure 2A**). The Red Face variety cultivated in Zhaotong (ZT99), resulted in a significant increase in the levels of various health-promoting flavonoids, than those found in the native ZT strawberry variety. Also, these results suggest that the altitude-associated ecological factors play a crucial role in modulating flavonoids biosynthesis in strawberry fruits.

The PCA of flavonoid varieties revealed that the PC1 accounted for 54.3% of the total variance along the horizontal axis, while the PC2 explained 24% of the variance on vertically axis (**Figure 2B**). In the PCA plot, the ZT variety clustered in the upper-left quadrant, DD99 was positioned on the right side near to the horizontal axis, and ZT99 was located in the lower-left region (**Figure 2B**). A separate PCA focusing on individual compounds demonstrated that the majority of flavonoids clustered near the origin, with the exception of six distinct compounds (**Figure 2C**). Among these, four were situated adjacent to the main cluster, while two were notably distant and contributed significant variation in the PCA plot (**Figure 2C**). In this analysis, PC1 represented 74% of the variance along the x-axis, and PC2 accounted for 25.6% of the variance on the y-axis (**Figure 2C**). The observed variations in flavonoid profiles among the strawberries demonstrate the significant impact of diverse altitude-associated ecological conditions on the biosynthesis and accumulation of these bioactive compounds within strawberry fruits.

### 3.3 Flavonoids fold changed into strawberry fruits

Among the 163 identified flavonoids, 115 flavonoids exhibited more than a two-fold change, either up-regulated or down-regulated, across distinct strawberry varieties (**Table 1**). The results indicate that 55 flavonoids were more than two-fold higher in ZT99 samples compared to DD99, while 30 flavonoids were more than two-fold down-regulated in ZT99 relative to DD99 samples (**Table 1**). Furthermore, 34 flavonoids were found to be elevated (more than 2-folds) in ZT99 samples compared to ZT, whereas 14 flavonoids were more than two-fold down-regulated in ZT99 samples compared to ZT (**Table 1**). Additionally, 43 flavonoids exhibited more than a two-fold increase in ZT compared to DD99, while 37 flavonoids were more than two-fold down-regulated in ZT compared to DD99 (**Table 1**). These results showed that the high-altitude-associated conditions of Zhaotong facilitate ZT99 fruits to accumulate more flavonoid compounds than the DD99 fruits grown at lower-altitude-associated conditions of Dandong.

**Table 1:** Flavonoids more than two-folds changed (FC) among strawberry fruits.

Serial No.	Compounds	FC higher in DD99 than ZT99	FC high in ZT99 than DD99	FC higher in ZT than ZT99	FC higher in ZT99 than ZT	FC higher in ZT than DD99	FC is higher in DD99 than ZT
1	2-hydroxynaringenin*	2.9	–	–	–	–	–
2	3’-methoxyquercetin-3-O-L-rhamnosyl(1→2)-glucopyranoside*	–	2.9	–	–	2.9	–
3	3,3’,4’,5’,7-Pentahydroxyflavone; Robinetin	–	2.6	–	–	2.4	–
4	F1	–	2.1	–	19.6	–	9.4
5	4’-O-Glucosylvitexin	–	–	–	3.5	–	–
6	Eucalyptin	9.7	–	3.0	–	–	3.2
7	6’’-O-Malonylisosakuranin	–	–	–	–	–	2.2
8	6-C-MethylKaempferol-3-glucoside*	–	2.6	–	–	2.1	–
9	6-Hydroxy-4’,5,7-trimethoxyflavanone; Hamiltone A	8.3	–	3.5	–	–	2.4
10	6-Hydroxykaempferol-3,6-O-Diglucoside*	4.5	–	–	–	–	7.5
11	6-Hydroxykaempferol-3,6-O-Diglucoside-7-O-Glucuronide	–	–	–	–	2.0	–
12	6-Hydroxykaempferol-6,7-O-Diglucoside*	36.3	–	7.2	–	–	5.0
13	6-Hydroxykaempferol-7,6-O-Diglucoside	3.8	–	–	7.7	–	29.2
14	6-Hydroxyluteolin 5-glucoside*	–	2.5	–	–	–	–
15	6-Methoxykaempferol-3-O-glucoside*	–	4.5	–	–	3.5	–
16	8-Methoxykaempferol-7-O-rhamnoside	–	2.3	–	–	4.5	–
17	Apigenin-5-O-glucoside	3.6	–	3.4	–	–	–
18	Apigenin-6-C-glucoside (Isovitexin)*	–	9.1	–	–	15.1	–
19	Apigenin-7-O-glucoside(Cosmosiin)	3.9	–	2.8	–	–	–
20	Apigenin-8-C-Glucoside (Vitexin)*	–	2.3	–	–	2.9	–
21	Aromadendrin (Dihydrokaempferol)	2.8	–	–	–	–	–
22	Avicularin(Quercetin-3-O-α-L-arabinofuranoside)	–	–	–	4.5	–	2.6
23	Azaleatin (5-O-Methylquercetin)	–	3.1	–	–	3.9	–
24	Buddlenoid A*	–	–	–	3.1	–	–
25	Carthamidin	2.7	–	–	–	–	–
26	Choerospondin	3.4	–	–	–	–	2.0
27	Chrysoeriol-7-O-glucoside	53.9	–	8.3	–	–	6.5
28	Cirsimaritin-8-C-[glucosyl-(1-2)]-glucoside	–	2.0	–	–	2.1	–
29	Cyrtominetin	–	2.8	2.0	–	5.5	–
30	Didymin (Isosakuranetin-7-O-rutinoside)*	–	–	–	2.5	–	–
31	Diosmetin-7-O-galactoside*	–	2.3	–	–	–	–
32	Diosmetin-7-O-glucuronide	–	2.7	–	–	3.3	–
33	Eriodictyol-7-O-(6’’-O-p-coumaroyl)glucoside	–	2.0	–	2.4	–	–
34	Eriodictyol-7-O-Rutinoside (Eriocitrin)	2.4	–	–	–	–	–
35	Eriodictyol-7-O-glucoside*	–	2.4	–	–	3.1	–
36	Galangin 7-glucoside	3.3	–	3.3	–	–	–
37	Gossypetin-7-O-L-rhamnopyranoside(Rhodiolgin)	–	–	–	–	3.1	–
38	Gossypetin-8-O-β-D-glucuronide	–	4.1	–	–	4.3	–
39	Hesperetin-5-O-glucoside	–	2.7	–	–	2.5	–
40	Hesperetin-7-O-glucoside	–	2.6	–	–	–	–
41	Hesperetin-7-O-neohesperidoside(Neohesperidin)	–	20.4	–	20.4	–	–
42	Hesperetin-7-O-rutinoside (Hesperidin)	–	8.5	–	10.5	–	–
43	Hispidulin-7-O-glucoside(Homoplantaginin)*	–	2.1	–	–	2.0	–
44	Isohemiphloin	–	3.1	–	–	3.7	–
45	Isookanin	3.0	–	–	–	–	–
46	Isoorientin-7-O-glucoside	2.3	–	–	–	–	2.8
47	Isorhamnetin-3-O-Glucoside*	–	3.1	–	–	2.8	–
48	Isorhamnetin-3-O-rutinoside (Narcissin)*	–	5.8	–	–	6.2	–
49	Isorhamnetin-7-O-glucoside (Brassicin)*	–	3.9	–	–	3.4	–
50	Kaempferol (3,5,7,4’-Tetrahydroxyflavone)	2.8	–	–	–	–	–
51	Kaempferol glucosyl dimalonyl glucoside	–	–	–	5.9	–	4.0
52	Kaempferol-3,7-O-diglucoside	2.2	–	–	–	–	2.8
53	Kaempferol-3,7-O-dirhamnoside (Kaempferitrin)	–	2.1	–	–	–	–
54	Kaempferol-3-O-(2’’-O-acetyl)glucoside	4.1	–	–	–	–	3.6
55	Kaempferol-3-O-(2’’-p-Coumaroyl)galactoside*	–	–	–	3.1	–	–
56	Kaempferol-3-O-(6’’-Malonyl)glucoside-7-O-Glucoside	–	–	–	3.6	–	4.2
57	Kaempferol-3-O-(6’’-O-acetyl)glucoside	–	–	–	–	–	2.6
58	Kaempferol-3-O-(6’’-malonyl)galactoside*	–	–	–	2.1	–	2.7
59	Kaempferol-3-O-(6’’-malonyl)glucoside*	–	–	–	–	–	2.6
60	Kaempferol-3-O-(6’’-p-Coumaroyl)galactoside*	–	–	–	3.5	–	2.0
61	Kaempferol-3-O-(6’’-p-Coumaroyl)glucoside (Tiliroside)	–	–	–	2.7	–	–
62	Kaempferol-3-O-arabinoside-7-O-rhamnoside	–	2.7	–	–	3.5	–
63	Kaempferol-3-O-glucorhamnoside*	3.1	–	–	–	–	2.6
64	Kaempferol-3-O-glucuronide-7-O-glucoside	–	–	–	2.9	–	2.8
65	Kaempferol-3-O-mannoside (Amoenin)	–	–	4.7	–	3.9	–
66	Kaempferol-3-O-robinobioside(Biorobin)*	2.2	–	–	–	–	–
67	Kaempferol-3-O-rutinoside(Nicotiflorin)*	4.9	–	–	4.2	–	20.4
68	Kaempferol-3-O-sophoroside	2.8	–	–	–	–	3.6
69	Ladanetin-6-O-β-D-glucoside*	–	2.4	–	–	2.6	–
70	Laricitrin-3-O-glucoside*	–	4.9	–	8.8	–	–
71	Liquiritigenin-4’-O-Glucoside (Liquiritin)	–	–	–	–	–	2.1
72	Luteolin-6-C-(2’’-glucuronyl)glucoside	–	–	–	4.5	–	3.6
73	Luteolin-7-O-(6’’-eudesmyl)glucoside	–	–	–	2.2	–	2.8
74	Luteolin-7-O-(6’’-malonyl)glucoside*	–	–	–	–	–	2.4
75	Luteolin-7-O-gentiobioside	2.6	–	–	–	–	4.0
76	Luteolin-7-O-neohesperidoside (Lonicerin)*	–	–	–	2.1	–	3.1
77	Luteolin-7-O-rutinoside*	2.8	–	–	–	–	3.3
78	Myricetin-3,7,3’-trimethyl ether	–	2.1	–	3.2	–	–
79	Myricetin-3-O-glucuronide	–	–	2.5	–	3.2	–
80	Myricetin-3-O-β-D-glucoside	–	3.4	–	–	4.2	–
81	Naringenin-4’-O-glucoside*	–	2.0	–	–	–	–
82	Naringenin-7-O-(6’’-malonyl)glucoside	–	–	–	–	3.1	–
83	Naringenin-7-O-Neohesperidoside(Naringin)	–	–	–	2.1	–	–
84	Nepetin-7-O-alloside	–	4.0	–	–	3.4	–
85	Norartocarpetin	3.0	–	–	–	–	–
86	Patuletin-3-O-glucoside*	–	5.2	–	3.7	–	–
87	Persicoside	–	–	2.0	–	2.7	–
88	Pinocembrin-7-O-neohesperidoside*	–	–	–	2.2	–	–
89	Poncirin (Isosakuranetin-7-O-neohesperidoside)*	–	–	–	2.4	–	–
90	Quercetin 3-O-glucoside 7-O-xyloside	–	6.1	2.2	–	13.1	–
91	Quercetin-3-O-(2’’-O-glucosyl)glucuronide	–	3.7	–	3.0	–	–
92	Quercetin-3-O-(4’’-O-glucosyl)rhamnoside*	–	3.1	–	–	2.8	–
93	Quercetin-3-O-(4’’-glucosyl)glucoside; Meratin	–	2.2	–	3.6	–	–
94	Quercetin-3-O-apiosyl(1→2)galactoside*	–	4.0	2.2	–	8.8	–
95	Quercetin-3-O-glucoside (Isoquercitrin)	–	2.8	–	–	–	–
96	Quercetin-3-O-glucoside-7-O-rhamnoside*	2.5	–	–	3.1	–	7.6
97	Quercetin-3-O-glucuronide	–	3.2	–	–	2.9	–
98	Quercetin-3-O-neohesperidoside*	2.7	–	–	2.0	–	5.4
99	Quercetin-3-O-robinobioside	8.8	–	–	–	–	8.8
100	Quercetin-3-O-rutinoside (Rutin)	–	–	–	3.2	–	5.6
101	Quercetin-3-O-sambubioside*	–	3.5	2.0	–	7.0	–
102	Quercetin-3-O-sambubioside-7-O-rhamnoside	–	6.9	–	–	4.0	–
103	Quercetin-4’-O-glucoside (Spiraeoside)*	–	2.6	–	–	–	–
104	Quercetin-4’-O-glucuronide*	–	2.1	–	–	2.1	–
105	Quercetin-5-O-glucuronide	–	2.6	–	–	2.4	–
106	Quercetin-5-O-β-D-glucoside*	–	3.0	–	–	2.2	–
107	Quercetin-7-O-glucoside	–	2.5	–	–	–	–
108	Quercetin-7-O-rutinoside*	2.0	–	–	2.2	–	4.6
109	Rhamnetin-3-O-Glucoside*	–	4.8	–	–	3.7	–
110	Tamarixetin-3-O-glucoside-7-O-rhamnoside*	–	17.7	–	–	15.4	–
111	Tamarixetin-3-O-rutinoside	–	5.4	–	–	6.1	–
112	Tricetin 3’-glucuronide*	–	2.2	–	–	2.2	–
113	Tricin-7-O-saccharic acid	–	–	–	2.1	–	2.2
114	Yuanhuanin*	–	2.9	–	–	3.4	–
115	xylosyl phellodendroside	–	2.3	–	19.4	–	8.5

* means isomers.

The 115 flavonoid compounds that were 2-fold or more than 2-fold altered among strawberry varieties (**Table 1**). The DD99 strawberry variety which is cultivated at lower-altitude showed that the chrysoeriol-7-O-glucoside 53.9, 6-hydroxykaempferol-6,7-O-diglucoside 36.3, eucalyptin 9.7, quercetin-3-O-robinobioside 8.8, and hamiltone A 8.3, were fold higher than ZT99 strawberry, which is cultivated at higher-altitude environment of Zhaotong (**Table 1**). The ZT99 strawberry showed that the neohesperidin 20.4, tamarixetin-3-O-glucoside-7-O-rhamnoside 17.7, isovitexin 9.1, hesperidin 8.5, tamarixetin-3-O-rutinoside 5.4, quercetin-3-O-sambubioside-7-O-rhamnoside 6.9, quercetin 3-O-glucoside 7-O-xyloside 6.1, narcissin 5.8, and patuletin-3-O-glucoside 5.2, were fold higher than DD99 strawberry variety (**Table 1**). This differential concentrations of flavonoids in response to varying environmental conditions, providing valuable insights into the metabolic adaptations of these strawberries.

### 3.4 Percentage of major flavonoids and their correlation with strawberry varieties

**Figure 3:**
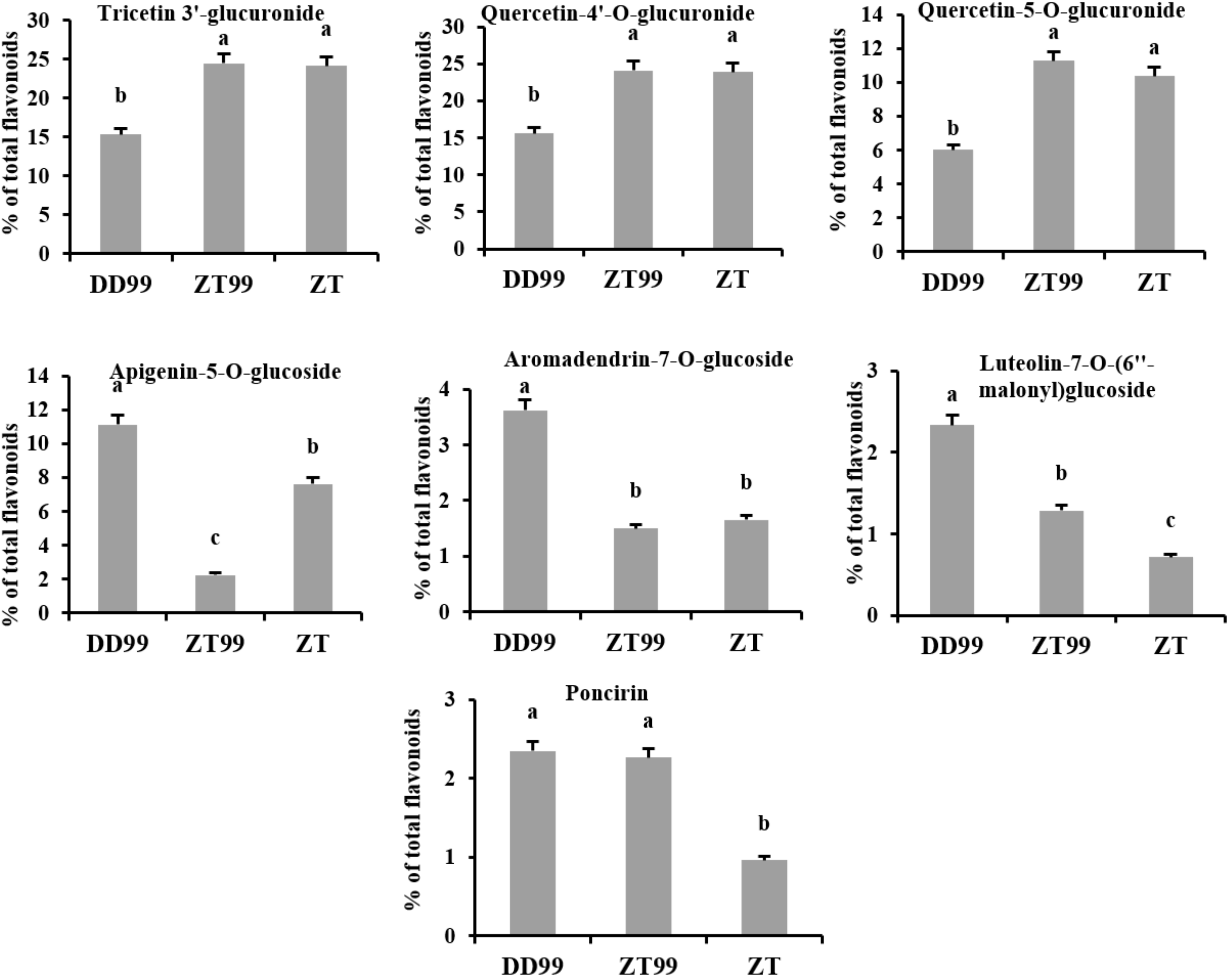
Percentage of major flavonoid compounds in the fruits of strawberry varieties. The data presented in each bar represents the mean of three biological replicates, with error bars indicating the standard error. Statistical analysis was conducted using the least significant difference (LSD) method to determine significant differences between fruits from various strawberry varieties, with significance level at p<0.05. Distinct letters (a–c) denote statistically significant differences among samples.

The relative abundance of major flavonoid compounds was calculated as a percentage of total flavonoids (**Figure 3**). Tricetin 3’-glucuronide exhibited the highest percentage, accounting for 24.49%, 24.14%, and 15.31% in ZT99, ZT, and DD99 samples, respectively. This was closely followed by quercetin-4’-O-glucuronide, which constituted 24.15%, 23.88%, and 15.59% in ZT99, ZT, and DD99 samples, respectively (**Figure 3**). These two compounds emerged as the predominant flavonoids in the strawberry fruits analyzed. Notably, the relative abundance of these compounds was significantly higher in fruits cultivated at higher altitudes (ZT99 and ZT) compared to those grown at lower elevations (DD99) (**Figure 3**). This observation suggests a potential correlation between altitude-associated ecological conditions and flavonoid accumulation in strawberry fruits. Similarly, the percentage of quercetin-5-O-glucuronide was significantly higher in ZT99 (11.26%) and ZT (10.36%) compared to DD99 (6.02%) (**Figure 3**). This trend further corroborates the hypothesis that altitude-associated ecological conditions may play a crucial role in modulating flavonoid biosynthesis and accumulation in strawberry fruits.

The concentrations of specific flavonoids such as apigenin-5-O-glucoside, aromadendrin-7-O-glucoside, and luteolin-7-O-(6’’-malonyl)glucoside were notably higher in DD99 (11.12%, 3.62%, and 2.34%, respectively) compared to ZT99 (2.24%, 1.5%, and 1.28%, respectively) and ZT (7.63%, 1.66%, and 0.71%, respectively) (**Figure 3**). These findings indicate that the Red Face variety, when cultivated at lower altitudes (DD99), exhibited elevated levels of these compounds. Conversely, their concentrations decreased significantly in samples of the same variety grown under higher-altitude (ZT99) ecological conditions in Zhaotong. Interestingly, poncirin levels did not show significant altitude-dependent variation between DD99 (2.35%) and ZT99 (2.27%); however, the ZT variety demonstrated a significantly lower poncirin concentration (0.96%) (**Figure 3**). These observed variations in flavonoid profiles suggest potential adaptive mechanisms in response to altitude-associated environmental factors. Such insights could prove valuable for breeding programs aimed at enhancing the nutritional and therapeutic value of strawberries.

**Figure 4:**
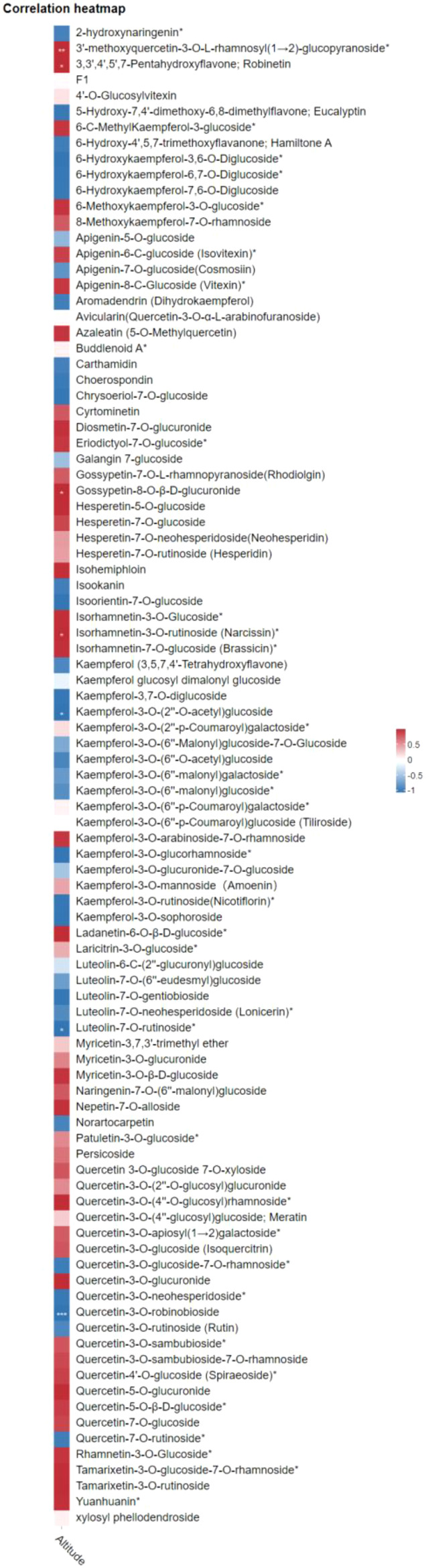
Pearson correlation heat-map of flavonoids with altitude. The red color shows positive correlation of flavonoid and with altitude and blue color signifies negative correlation of flavonoid with altitude. The “*” indicate significant correlation of flavonoids with altitude is significant.

A correlation heat map analysis was conducted to examine the relationship between flavonoid compounds and altitude (**Figure 4**). The analysis revealed distinct distribution patterns of flavonoid metabolites, with 36 compounds showing positive correlations with elevation (indicated in red) (**Figure 4**). Among these positively correlated compounds, four flavonoids - 3’-methoxyquercetin-3-O-L-rhamnosyl(1→2)-glucopyranoside, robinetin, gossypetin-8-O-β-D-glucuronide, and narcissin - demonstrated particularly strong and statistically significant correlations. These compounds exhibited consistent altitude-dependent accumulation patterns, with higher concentrations observed in strawberry samples harvested from the high-altitude ecological conditions of Zhaotong compared to the lower-altitude conditions of Dandong.

Conversely, 29 flavonoid compounds exhibited negative correlations with altitude, indicating an inverse relationship between their abundance and elevation. Among these, quercetin-3-O-robinobioside, kaempferol-3-O-(2’’-O-acetyl)glucoside, and luteolin-7-O-rutinoside demonstrated strong and significantly negative correlations (highlighted in blue color) (**Figure 4**). The concentrations of these metabolites showed a significant decrease in strawberry fruits harvested from the high-altitude ecological conditions of Zhaotong compared to the lower-altitude conditions of Dandong, suggesting adaptive responses to elevation-associated environmental conditions. These bidirectional correlation patterns provide valuable insights into how altitude-dependent ecological conditions influence the regulation of endogenous flavonoid metabolism.

### 3.5 Total metabolic contents and antioxidant capacity of strawberry fruits

**Figure 5:**
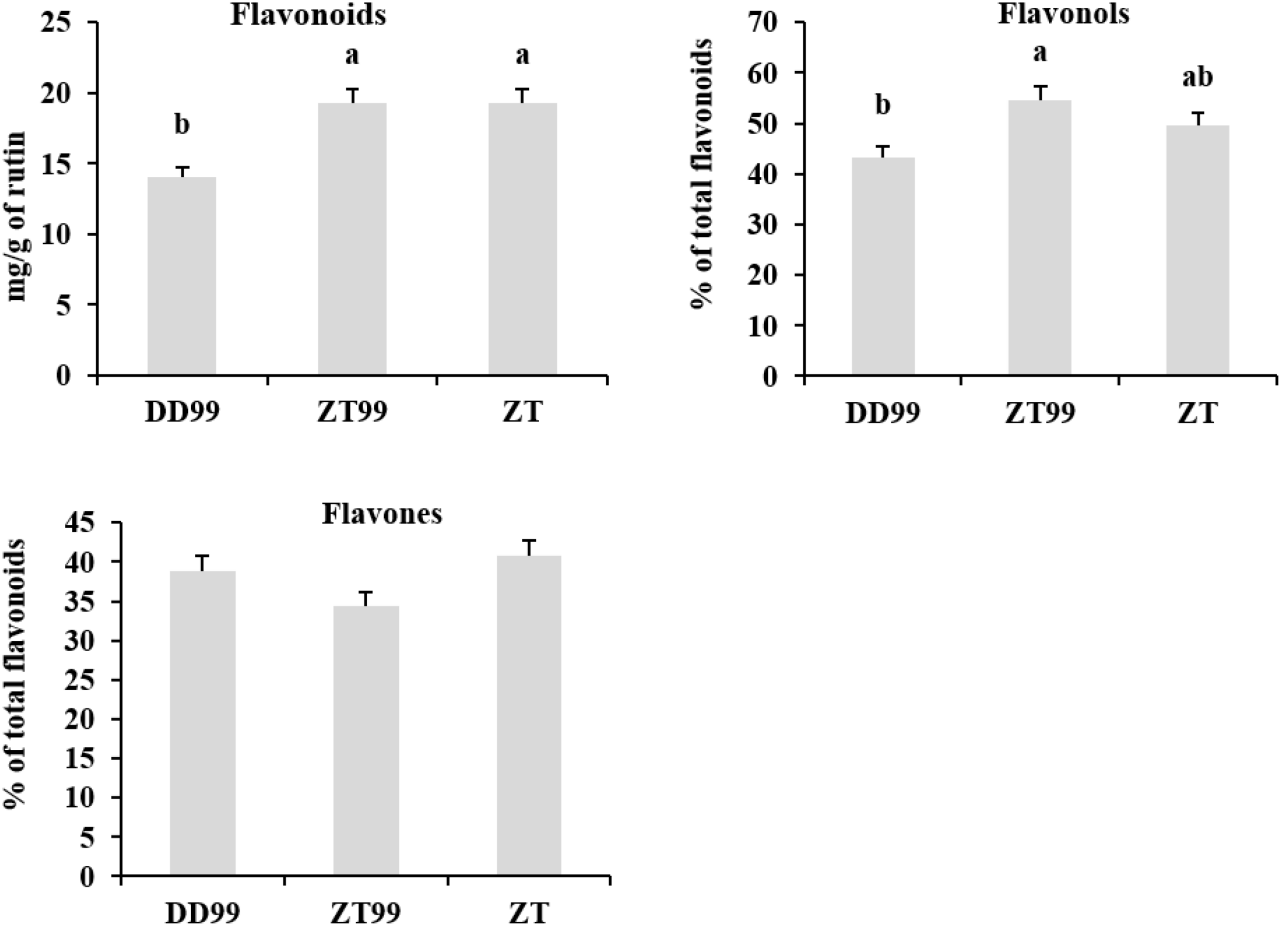
Total contents of flavonoids, flavonols, and flavones in three strawberry fruits. The data presented in each bar represents the mean of three biological replicates, with error bars indicating the standard error. Statistical analysis was conducted using the least significant difference (LSD) method to determine significant differences between fruits from various strawberry varieties, with significance level at p<0.05. Distinct letters (a–c) denote statistically significant differences among samples.

The ZT and ZT99 strawberries exhibited significantly higher total flavonoid contents (19.28 mg/g and 19.26 mg/g of rutin, respectively) compared to DD99 (14.05 mg/g of rutin) (**Figure 5**). The ZT99 strawberries demonstrated a significantly higher percentage of flavonols (54.61%), followed by ZT (49.65%), while DD99 exhibited a significantly lower percentage of flavonol content (43.37%) (**Figure 5**). These findings indicate that the “Red Face” strawberries harvested from higher-altitudes (ZT99) conditions of Zhaotong significantly enhances total flavonols accumulation compared to samples harvested from lower-altitudes conditions (DD99) of Dandong. Regarding flavones, ZT displayed a higher percentage (40.75%), followed by DD99 (38.88%), whereas ZT99 strawberry fruits showed lower percentage of flavones (34.45%); however, the flavone variations among strawberry samples were insignificant (**Figure 5**).

**Figure 6:**
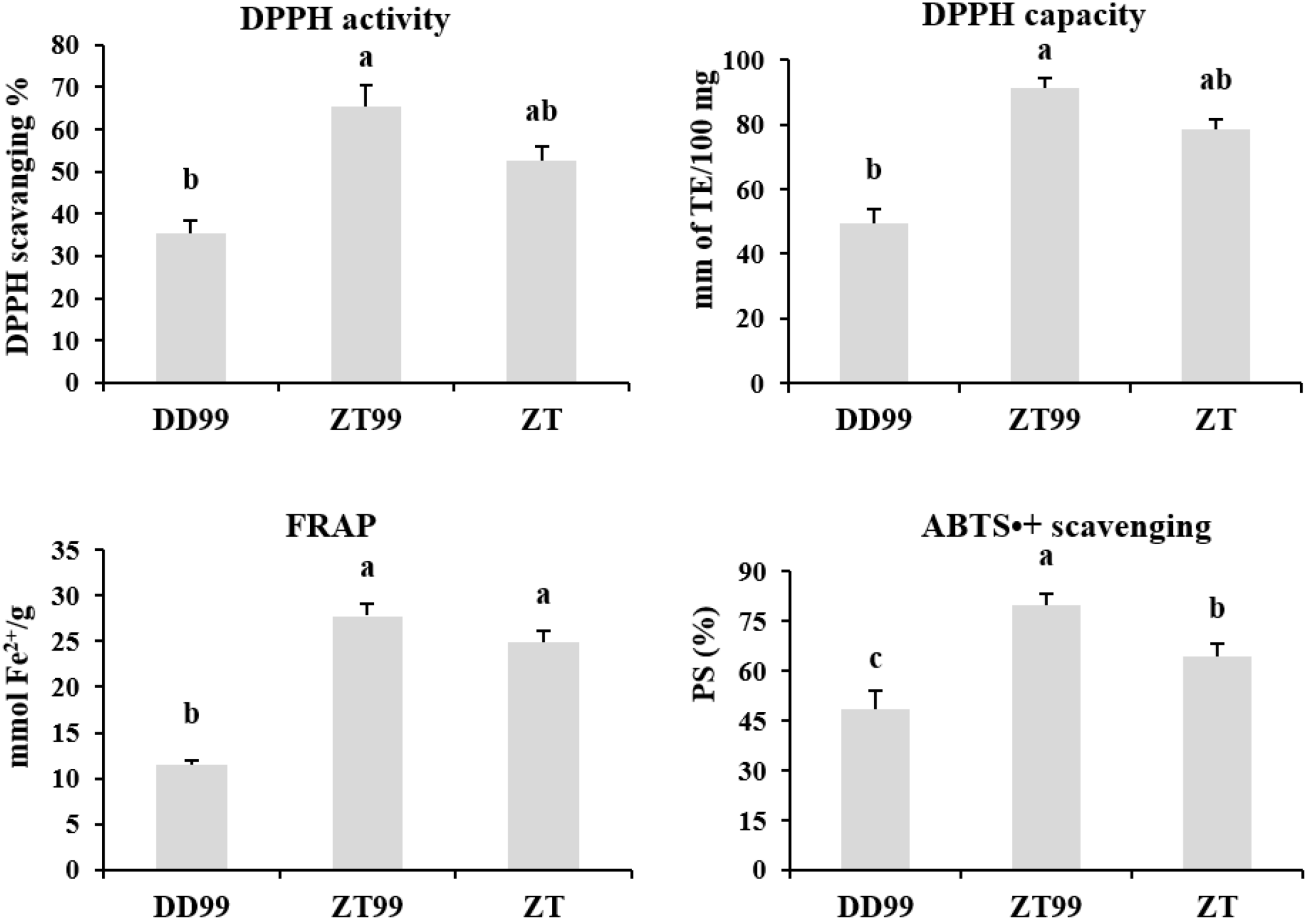
Antioxidant activity and capacity of flavonoids in strawberry fruits cultivated under diverse environmental conditions. The data presented in each bar represents the mean of three biological replicates, with error bars indicating the standard error. Statistical analysis was conducted using the least significant difference (LSD) method to determine significant differences between fruits from various strawberry varieties, with significance level at p<0.05. Distinct letters (a–c) denote statistically significant differences among samples.

Antioxidant profiling was conducted to evaluate the antioxidant potential and free radical scavenging capacities of strawberry fruits using multiple assays (**Figure 6**). The “Red Face” strawberry samples obtained from the high-altitude region of Zhaotong (ZT99) demonstrated superior antioxidant properties across all antioxidant assays. Specifically, ZT99 samples exhibited enhanced DPPH activity of 65.34% and antioxidant capacity of 91.39 mm TE/100 mg, followed by ZT samples. In contrast, “Red Face” strawberry samples from Dandong (DD99) exhibited significantly lower DPPH activity (35.47%) and antioxidant capacity (49.46 mm TE/100 mg). Furthermore, FRAP analysis revealed elevated reducing capacity of 27.76 mmol Fe^2+^/g and ABTS•+ radical cation scavenging activity of 79.92 PS% in ZT99 samples. DD99 samples, however, demonstrated significantly reduced antioxidant capabilities, with FRAP values of 11.45 mmol Fe^2+^/g and ABTS•+ scavenging activity of 48.28 PS% (**Figure 6**). These findings suggest that the cultivation of the Red Face variety under high-altitude environmental conditions in Zhaotong resulted in significant increment of antioxidant scavenging activities, potentially attributed to environmental responses and adaptive mechanisms induced by high-altitude ecological conditions.

### 3.6 Strawberry fruit quality attributes and correlation analysis

Strawberry fruit quality attributes assessment was conducted to evaluate key physicochemical parameters, including soluble solids content (SSC), ascorbic acid concentration, titratable acidity, and fruit firmness (**Table 2**). The ZT99 fruit samples demonstrated significantly superior quality characteristics, particularly regarding SSC (15.30°Brix) and ascorbic acid content (15.73 mg/g), compared to DD99 fruit samples, which exhibited markedly lower values of 10.96°Brix and 8.53 mg/g, respectively (**Table 2**). Notably, the ZT strawberry samples showed reduced levels of ascorbic acid (7.82 mg/g), titratable acidity (0.31%), and fruit firmness (8.46 N) (**Table 2**). No significant variations in titratable acidity and firmness were observed between DD99 and ZT99 samples. Comparative assessment of the “Red Face” strawberry variety under contrasting ecological conditions demonstrated that high-altitude cultivation significantly enhanced both soluble solid content and ascorbic acid synthesis. Specifically, fruits harvested from the ZT99 high-altitude site exhibited higher levels of these compounds compared to those from the DD99 low-altitude environments in Dandong (**Table 2**). These findings exhibit the substantial influence of ecological conditions on the development of key quality attributes in strawberry fruits.

**Table 2:** Quality attributes of strawberry fruits.

Serial No.	Sample names of Varieties	SSC (°Brix)	Ascorbic acid(mg/g)	Titratable acid (%)	Firmness(N)
1	DD99	10.96 ± 0.84 ^b^	8.53 ± 1.73 ^b^	0.45 ± .02 ^a^	23.16 ± 1.04 ^a^
2	ZT99	15.30 ± 1.23 ^a^	15.73 ± 1.51 ^a^	0.41 ± .03 ^a^	20.51 ± 0.96 ^a^
3	ZT	13.23 ± 1.06 ^ab^	7.82 ± 1.72 ^b^	0.31 ± .01 ^b^	8.46 ± 0.59 ^b^

SSC, soluble solids content.

Superscript letters means significantly different.

The phytochemical correlation analysis revealed complex interconnections among diverse bioactive compounds and quality parameters. Total concentrations of flavonoids, flavonols, and flavones demonstrated statistically significant positive correlations with all variables, except the ascorbic acid, titratable acid, and firmness (**Table 3**). This observation suggests coordinated biosynthetic pathways among these flavonoid compounds. All antioxidant scavenging assays exhibited statistically significant positive correlations with one another, as well as with flavonoids, flavonols, and sugars (**Table 3**), indicating these metabolites’ substantial contribution to the samples’ overall antioxidant capacity. Ascorbic acid exhibited non-significant positive correlations with all variables except flavones, where a slight negative correlation was observed (**Table 3**). Titratable acid and firmness demonstrated negative correlations with all variables except ascorbic acid. Notably, titratable acid and ascorbic acid displayed a significant positive correlation (**Table 3**), suggesting potential shared regulatory mechanisms or complementary roles in fruit acid metabolism. These findings provide insights into the intricate interactions between strawberry fruits quality parameters and their bioactive compounds, which may have significant implications for future breeding programs and postharvest management strategies.

**Table 3:** Strawberry fruits correlation analysis among flavonoids and antioxidant assays.

Variables	FLA	Flavonol	Flavone	DPPH A	DPPH C	FRAP	ABTS	Sugar	ASA	TA	FN
Flavonoid	1										
Flavonol	0.97^a^	1									
Flavone	0.86 ^a^	0.71	1								
DPPH A	0.90 ^a^	0.98 ^a^	0.56	1							
DPPH C	0.95 ^a^	0.99 ^a^	0.67	0.99 ^a^	1						
FRAP	0.99 ^a^	0.99 ^a^	0.76	0.96 ^a^	0.99 ^a^	1					
ABTS	0.87 ^a^	0.96 ^a^	0.50	0.99 ^a^	0.98 ^a^	0.94 ^a^	1				
Sugar	0.97 ^a^	0.99 ^a^	0.72	0.98 ^a^	0.99 ^a^	0.99 ^a^	0.96 ^a^	1			
ASA	0.43	0.64	-0.09	0.78	0.68	0.57	0.82	0.63	1		
TA	-0.62	-0.41	-0.93 ^a^	-0.22	-0.35	-0.48	-0.15	-0.41	0.45	1	
FN	-0.71	-0.52	-0.97 ^a^	-0.34	-0.46	-0.58	-0.27	-0.52	0.33	0.99 ^a^	1

a Significant and positive correlation, FLA, Flavonoid; DPPH A, DPPH activity; DPPH C, DPPH capacity; ASA, Ascorbic acid; TA, Titratable acid; FN means firmness.

## 4 Discussion

Bioactive secondary metabolites such as phenolics, flavonoids, flavones, and flavonols are widely distributed in plant Kingdom, including fruits, flowers, vegetables, and cereal crops (Khoo et al., 2017). These bioactive secondary metabolites are regulated by various abiotic (including heat, cold, light intensity, UV light, drought stress, water logging, salinity, and nutrient availability) and biotic (pathogen infection, herbivory, etc.) factors, which play crucial roles in their biosynthesis, accumulation, and metabolism within plants (Rao et al., 2018, 2022a, 2022b). Previous studies indicated that cultivation of plants at different locations can significantly influence the phytochemical composition of strawberry fruits (Josuttis et al., 2013). Here, we have identified 163 bioactive flavonoids (**Supplementary Table S1**), in strawberry fruits cultivated in varying ecological conditions. Using LC-MS/MS analysis, we observed that altitude-associated environmental conditions significantly influenced the flavonoid profiles of strawberries. Previous research states that higher altitudes-associated environmental conditions cause significant variations in the biosynthesis of bioactive secondary metabolites, such as phenolics, flavonoids, anthocyanins (Feng et al., 2017).

A total number of 115 bioactive flavonoid compounds were more than 2-folds changed in the strawberry fruits cultivated in high- and low-altitude-related environmental conditions (**Table 1**). Different altitudes-based environmental factors such as sunlight exposure, light/dark interval, UV radiation, and temperature variations cause significant impact on the plants primary and secondary metabolism (Carbone et al., 2009). Among 115 bioactive flavonoids, 55 flavonoids were more than two-fold higher in ZT99 samples compared to DD99, whereas the 30 flavonoids were higher in DD99 than ZT99 samples (**Table 1**). These findings indicate that altitude-related environmental factors play a crucial role in the regulation of bioactive metabolites of strawberry fruits. Environmental stress at high-altitudes triggers the plant’s secondary metabolic pathways and contributes to enhancement of plant antioxidant activities (Josuttis et al., 2013), suggests that the plants cultivated at higher-altitudes have more potential health benefits than the plants grown at lower-altitudes (Dzhanfezova et al., 2020; Rao et al., 2021).

Notably, the ZT99 strawberry samples showed that the neohesperidin, isovitexin, and hesperidin were 20.4-, 9.1-, 8.5-fold higher than the DD99 strawberry samples grown at lower-altitude (**Table 1**). These three bioactive flavonoids have strong free radical quenching activity and help plants to adapt different environmental conditions (Rao et al., 2023, 2024b, 2024a). Earlier research revealed that environmental factors had significant effect on the flavonoids and anthocyanins accumulation and composition in fruits (Kalt et al., 2001). Increased UV radiation and lower temperatures stimulates flavonoids and anthocyanins accumulation in plants (Santos-Buelga and Scalbert, 2000; Buendia et al., 2010; Rao et al., 2019; Shi et al., 2023). Neohesperidin is famous due to its potent antioxidant activity in plants as well as in humans and protects the cellular DNA damage, protein degradation and cell death (Choi et al., 2007). Hesperidin, a compound not synthesized by the human body, demonstrates multiple beneficial effects on human health when consumed through plant-based sources. This bioflavonoid has been shown to reduce cardiovascular disease risk factors and exhibits significant neuroprotective properties. Furthermore, hesperidin shows promise as an anticancer agent and plays a crucial role in central nervous system function (Devi et al., 2015; Hajialyani et al., 2019; Mas-Capdevila et al., 2020; Rui et al., 2024). Vitexin and isovitexin compounds have resilient pharmacological value and are used as promising therapeutic agents against cancer therapy (Ganesan and Xu, 2017). Our findings suggest the existence of complex regulatory mechanisms that modulate the biosynthesis and accumulation of specific flavonoid compounds in response to environmental conditions associated with different altitudes.

Flavonoid glucuronides are known for their biological activities, free radical scavenging, anti-inflammatory, antibacterial activities and some glucuronides have anti-tumor activity and have positive effects on cardiocerebrovascular diseases (Chen et al., 2022). Our results showed that the ZT99 strawberry fruits have a significantly higher percentage of tricetin 3’-glucuronide 24.49%, quercetin-4’-O-glucuronide 24.15%, and quercetin-5-O-glucuronide 11.26% than DD99 strawberry fruits 15.31%, 15.59%, and 6.02%, respectively (**Figure 3**). The quercetin 3’-glucuronide has strong anti-tumor activity against human breast cancer MCF-7 cells (Wu et al., 2018). Among various flavonoid derivatives, glucuronides are interesting due to their enhanced bioavailability and therapeutic potential (Chen et al., 2022). Our findings showed that cultivation of the Red Face strawberry under high-altitude environmental conditions in Zhaotong results in significantly enhanced bioactive flavonoids, compared to fruits of the same variety grown at lower elevations (DD99) in Dandong. These differences in key flavonoid compounds demonstrate a clear altitude-dependent effect on secondary metabolite production. The identification of these altitude-responsive flavonoids contributes to our understanding of plant metabolic plasticity and adaptation mechanisms across elevation gradients.

According to our results, the sugar and ascorbic acid contents were notably increased in the ZT99 fruit samples with enhanced antioxidant activity and capacity than DD99 fruit samples (**Figure 6**, **Table 2**). The quality attributes of strawberries are crucial determinants of consumer acceptance and market value. Among these, higher soluble sugars and increased ascorbic acid content are considered key parameters that define strawberry fruit as good quality (Cordenunsi et al., 2003; Giampieri et al., 2012; Pedrozo et al., 2023). The antioxidant properties of strawberries have gained considerable attention due to their potential role in preventing oxidative stress-related diseases and promoting human health. These antioxidant compounds can effectively neutralize free radicals and reduce cellular damage, potentially lowering the risk of various chronic diseases including cancer and cardiovascular disorders (Andersen and Markham, 2005; Kamboh et al., 2015; Shen et al., 2022). The marked difference in sugars, ascorbic acid, and antioxidant profiles between ZT99 and DD99 samples underscores the significant impact of environmental conditions on the biosynthesis and accumulation of sugars and antioxidant compounds in strawberry fruits. These findings suggest that high-altitude cultivation could be an effective strategy for enhancing the local strawberry production and the nutritional and functional properties of strawberry varieties compared to their native lower altitude growing conditions of Dandong.

## 5 Conclusions

Our research concluded that higher-altitude conditions in Zhaotong significantly improved the flavonoid contents, enhanced antioxidant activity, and quality attributes of the Red Face strawberry variety compared to lower-altitude grown fruits of same variety. Our analysis revealed significant alterations in flavonoid profiles, with 115 out of 163 identified compounds showing more than two-fold changes between lower- and higher-altitude associated cultivation sites. Notably, fruits grown in Zhaotong exhibited significantly higher levels of bioactive flavonoids such as neohesperidin, tamarixetin-3-O-glucoside-7-O-rhamnoside, and hesperidin, along with improved sugar content and ascorbic acid levels compared to fruits grown in Dandong. These findings have significant implications for commercial strawberry production, suggesting that strategic selection of cultivation altitude could be an effective approach to optimize both nutritional value and fruit quality. This research not only contributes to our understanding of environmental influences on strawberry metabolites but also offers practical insights for optimizing cultivation conditions to produce superior quality fruits with enhanced nutritional value. Future research should focus on the molecular mechanisms underlying these altitude-dependent variations and their potential application in breeding programs aimed at enhancing strawberry fruit characteristics.

## Data Availability

The original contributions presented in the study are included in the article/**Supplementary Material**. Further inquiries can be directed to the corresponding authors.

## Supplementary material

Supplementary Table 1 Illustrates the details of flavonoid compounds identified in this study.
